# Supplementary material for: Modeling glioblastoma heterogeneity as a dynamic network of cell states
Source: Mol Syst Biol. 2021 Sep 16;17(9):e10105. doi: 10.15252/msb.202010105 (PMC8444284; doi:10.15252/msb.202010105)
Supplement: Supplementary file 5 — Source Data for Figure 3 [file MSB-17-e10105-s001.zip › Figure3A_sourcedata/GSEA_3065/nosigdb_state2.GseaPreranked.1622718419215/RICHARDS_NOWAKOWSKI_SCIENCE_2017_ASTROCYTE_UPREG.html]

Details for gene set RICHARDS\_NOWAKOWSKI\_SCIENCE\_2017\_ASTROCYTE\_UPREG[GSEA]

|  || Dataset | state2 |
| Phenotype | NoPhenotypeAvailable |
| Upregulated in class | na\_neg |
| GeneSet | RICHARDS\_NOWAKOWSKI\_SCIENCE\_2017\_ASTROCYTE\_UPREG |
| Enrichment Score (ES) | -0.5504041 |
| Normalized Enrichment Score (NES) | -2.5266025 |
| Nominal p-value | 0.0 |
| FDR q-value | 0.0 |
| FWER p-Value | 0.0 |
Table: GSEA Results Summary

  

Fig 1: Enrichment plot: RICHARDS\_NOWAKOWSKI\_SCIENCE\_2017\_ASTROCYTE\_UPREG      
 Profile of the Running ES Score & Positions of GeneSet Members on the Rank Ordered List

  

| PROBE | GENE SYMBOL | GENE\_TITLE | RANK IN GENE LIST | RANK METRIC SCORE | RUNNING ES | CORE ENRICHMENT || 1 | CDCA7L |  |  | 167 | 0.301 | -0.0125 | No |
| 2 | SLC25A5 |  |  | 168 | 0.299 | -0.0064 | No |
| 3 | OLIG2 |  |  | 179 | 0.294 | -0.0016 | No |
| 4 | SRI |  |  | 181 | 0.293 | 0.0043 | No |
| 5 | DBI |  |  | 251 | 0.252 | 0.0017 | No |
| 6 | PHGDH |  |  | 271 | 0.243 | 0.0046 | No |
| 7 | LHX2 |  |  | 329 | 0.223 | 0.0028 | No |
| 8 | LDHB |  |  | 343 | 0.219 | 0.0058 | No |
| 9 | GNG5 |  |  | 389 | 0.207 | 0.0049 | No |
| 10 | RHOA |  |  | 442 | 0.192 | 0.0030 | No |
| 11 | AIF1L |  |  | 481 | 0.183 | 0.0025 | No |
| 12 | HNRNPA2B1 |  |  | 510 | 0.178 | 0.0031 | No |
| 13 | PSAT1 |  |  | 561 | 0.170 | 0.0009 | No |
| 14 | RHOBTB3 |  |  | 612 | 0.162 | -0.0014 | No |
| 15 | TRIB2 |  |  | 636 | 0.158 | -0.0007 | No |
| 16 | S100B |  |  | 688 | 0.151 | -0.0033 | No |
| 17 | FAM181B |  |  | 696 | 0.151 | -0.0010 | No |
| 18 | ID4 |  |  | 705 | 0.150 | 0.0011 | No |
| 19 | ENO1 |  |  | 717 | 0.148 | 0.0029 | No |
| 20 | GCSH |  |  | 723 | 0.148 | 0.0054 | No |
| 21 | MT2A |  |  | 731 | 0.146 | 0.0076 | No |
| 22 | FABP5 |  |  | 796 | 0.138 | 0.0033 | No |
| 23 | FABP7 |  |  | 810 | 0.137 | 0.0046 | No |
| 24 | PRTFDC1 |  |  | 818 | 0.137 | 0.0066 | No |
| 25 | SNX3 |  |  | 823 | 0.136 | 0.0089 | No |
| 26 | NEK6 |  |  | 839 | 0.134 | 0.0100 | No |
| 27 | UGP2 |  |  | 851 | 0.132 | 0.0114 | No |
| 28 | SPRY2 |  |  | 870 | 0.131 | 0.0121 | No |
| 29 | CREM |  |  | 873 | 0.131 | 0.0145 | No |
| 30 | IDI1 |  |  | 878 | 0.130 | 0.0167 | No |
| 31 | SNX5 |  |  | 942 | 0.125 | 0.0122 | No |
| 32 | SOX8 |  |  | 945 | 0.124 | 0.0145 | No |
| 33 | SOX2 |  |  | 1036 | 0.117 | 0.0069 | No |
| 34 | OLIG1 |  |  | 1090 | 0.112 | 0.0032 | No |
| 35 | TGIF1 |  |  | 1150 | 0.108 | -0.0011 | No |
| 36 | GNG12 |  |  | 1154 | 0.108 | 0.0007 | No |
| 37 | BAALC |  |  | 1158 | 0.107 | 0.0026 | No |
| 38 | MRFAP1 |  |  | 1167 | 0.107 | 0.0038 | No |
| 39 | SMC5 |  |  | 1172 | 0.106 | 0.0056 | No |
| 40 | CXXC5 |  |  | 1185 | 0.105 | 0.0064 | No |
| 41 | EPB41L2 |  |  | 1200 | 0.105 | 0.0069 | No |
| 42 | DDAH1 |  |  | 1260 | 0.101 | 0.0024 | No |
| 43 | DUSP6 |  |  | 1345 | 0.095 | -0.0050 | No |
| 44 | PDXK |  |  | 1353 | 0.095 | -0.0039 | No |
| 45 | NDUFB3 |  |  | 1444 | 0.090 | -0.0121 | No |
| 46 | CLNS1A |  |  | 1445 | 0.090 | -0.0103 | No |
| 47 | SOX9 |  |  | 1466 | 0.088 | -0.0107 | No |
| 48 | VEGFB |  |  | 1490 | 0.087 | -0.0115 | No |
| 49 | WSCD1 |  |  | 1616 | 0.081 | -0.0238 | No |
| 50 | LITAF |  |  | 1685 | 0.078 | -0.0298 | No |
| 51 | PRDX1 |  |  | 1763 | 0.074 | -0.0369 | No |
| 52 | JUN |  |  | 1805 | 0.072 | -0.0400 | No |
| 53 | TJP1 |  |  | 1811 | 0.072 | -0.0391 | No |
| 54 | LARS |  |  | 1838 | 0.071 | -0.0406 | No |
| 55 | ADI1 |  |  | 1839 | 0.071 | -0.0391 | No |
| 56 | PDLIM3 |  |  | 1841 | 0.071 | -0.0378 | No |
| 57 | ETV5 |  |  | 1843 | 0.071 | -0.0365 | No |
| 58 | C3orf70 |  |  | 1976 | 0.065 | -0.0499 | No |
| 59 | RAB31 |  |  | 1977 | 0.065 | -0.0485 | No |
| 60 | ZEB1 |  |  | 1995 | 0.065 | -0.0491 | No |
| 61 | TOMM7 |  |  | 1996 | 0.065 | -0.0478 | No |
| 62 | MPC1 |  |  | 2005 | 0.064 | -0.0474 | No |
| 63 | FAM120A |  |  | 2030 | 0.063 | -0.0488 | No |
| 64 | USP46 |  |  | 2090 | 0.061 | -0.0541 | No |
| 65 | CA2 |  |  | 2111 | 0.061 | -0.0551 | No |
| 66 | PEX2 |  |  | 2116 | 0.061 | -0.0543 | No |
| 67 | MAP3K1 |  |  | 2140 | 0.060 | -0.0557 | No |
| 68 | PEA15 |  |  | 2161 | 0.059 | -0.0567 | No |
| 69 | ZNF639 |  |  | 2195 | 0.058 | -0.0592 | No |
| 70 | RHOC |  |  | 2261 | 0.055 | -0.0654 | No |
| 71 | SEPT7 |  |  | 2302 | 0.054 | -0.0687 | No |
| 72 | ZFP36L1 |  |  | 2320 | 0.053 | -0.0695 | No |
| 73 | CNN3 |  |  | 2349 | 0.052 | -0.0716 | No |
| 74 | ADD3 |  |  | 2401 | 0.051 | -0.0763 | No |
| 75 | POLR2L |  |  | 2435 | 0.050 | -0.0789 | No |
| 76 | RHBDD2 |  |  | 2451 | 0.049 | -0.0796 | No |
| 77 | IMPACT |  |  | 2457 | 0.049 | -0.0792 | No |
| 78 | NDUFA4 |  |  | 2462 | 0.049 | -0.0786 | No |
| 79 | SCCPDH |  |  | 2482 | 0.048 | -0.0798 | No |
| 80 | WDR41 |  |  | 2484 | 0.048 | -0.0789 | No |
| 81 | SRGAP2B |  |  | 2503 | 0.047 | -0.0799 | No |
| 82 | MT1X |  |  | 2538 | 0.046 | -0.0828 | No |
| 83 | MT3 |  |  | 2560 | 0.046 | -0.0842 | No |
| 84 | SEPT2 |  |  | 2582 | 0.045 | -0.0856 | No |
| 85 | MAPK1 |  |  | 2595 | 0.045 | -0.0860 | No |
| 86 | PCCB |  |  | 2597 | 0.045 | -0.0852 | No |
| 87 | ARHGEF26 |  |  | 2624 | 0.044 | -0.0873 | No |
| 88 | HSDL2 |  |  | 2644 | 0.043 | -0.0885 | No |
| 89 | LAP3 |  |  | 2647 | 0.043 | -0.0879 | No |
| 90 | ITPK1 |  |  | 2684 | 0.042 | -0.0910 | No |
| 91 | FHL1 |  |  | 2686 | 0.042 | -0.0903 | No |
| 92 | ZCCHC24 |  |  | 2705 | 0.041 | -0.0914 | No |
| 93 | IL17RB |  |  | 2712 | 0.041 | -0.0913 | No |
| 94 | NDUFB5 |  |  | 2774 | 0.040 | -0.0973 | No |
| 95 | ANXA5 |  |  | 2812 | 0.039 | -0.1006 | No |
| 96 | C1orf61 |  |  | 2879 | 0.037 | -0.1072 | No |
| 97 | ALDH6A1 |  |  | 2912 | 0.036 | -0.1101 | No |
| 98 | THG1L |  |  | 2920 | 0.036 | -0.1101 | No |
| 99 | GNG7 |  |  | 2967 | 0.035 | -0.1145 | No |
| 100 | HIPK2 |  |  | 3029 | 0.033 | -0.1207 | No |
| 101 | SUCLG2 |  |  | 3045 | 0.033 | -0.1217 | No |
| 102 | EFHD2 |  |  | 3081 | 0.032 | -0.1249 | No |
| 103 | TSPAN3 |  |  | 3103 | 0.032 | -0.1266 | No |
| 104 | IFI27L1 |  |  | 3114 | 0.031 | -0.1271 | No |
| 105 | ZFP36L2 |  |  | 3122 | 0.031 | -0.1273 | No |
| 106 | PHLPP1 |  |  | 3141 | 0.031 | -0.1287 | No |
| 107 | RGCC |  |  | 3143 | 0.031 | -0.1281 | No |
| 108 | GAS1 |  |  | 3144 | 0.031 | -0.1275 | No |
| 109 | C2orf72 |  |  | 3151 | 0.031 | -0.1276 | No |
| 110 | PAX6 |  |  | 3226 | 0.029 | -0.1352 | No |
| 111 | ARHGAP5 |  |  | 3234 | 0.029 | -0.1354 | No |
| 112 | LIMA1 |  |  | 3279 | 0.028 | -0.1398 | No |
| 113 | PHYHIPL |  |  | 3290 | 0.028 | -0.1403 | No |
| 114 | PSMD10 |  |  | 3415 | 0.026 | -0.1536 | No |
| 115 | OGFRL1 |  |  | 3489 | 0.024 | -0.1613 | No |
| 116 | PURB |  |  | 3499 | 0.024 | -0.1618 | No |
| 117 | PPP2R5A |  |  | 3517 | 0.024 | -0.1632 | No |
| 118 | FAM19A5 |  |  | 3527 | 0.023 | -0.1637 | No |
| 119 | RASSF2 |  |  | 3560 | 0.023 | -0.1669 | No |
| 120 | MTSS1L |  |  | 3577 | 0.022 | -0.1682 | No |
| 121 | TLK1 |  |  | 3656 | 0.021 | -0.1765 | No |
| 122 | ZIC1 |  |  | 3668 | 0.021 | -0.1773 | No |
| 123 | GLUD1 |  |  | 3694 | 0.021 | -0.1796 | No |
| 124 | SLC48A1 |  |  | 3702 | 0.020 | -0.1800 | No |
| 125 | EFCAB2 |  |  | 3718 | 0.020 | -0.1813 | No |
| 126 | PARD3 |  |  | 3728 | 0.020 | -0.1819 | No |
| 127 | EFCAB14 |  |  | 3792 | 0.019 | -0.1885 | No |
| 128 | TACC1 |  |  | 3824 | 0.018 | -0.1916 | No |
| 129 | CRYL1 |  |  | 3830 | 0.018 | -0.1918 | No |
| 130 | WWC2 |  |  | 3889 | 0.017 | -0.1979 | No |
| 131 | UBL3 |  |  | 3891 | 0.017 | -0.1977 | No |
| 132 | ETV1 |  |  | 3908 | 0.017 | -0.1991 | No |
| 133 | RAB13 |  |  | 3928 | 0.017 | -0.2009 | No |
| 134 | FERMT2 |  |  | 3980 | 0.016 | -0.2063 | No |
| 135 | GSTM4 |  |  | 3990 | 0.016 | -0.2070 | No |
| 136 | OAT |  |  | 4004 | 0.015 | -0.2081 | No |
| 137 | SPATA6 |  |  | 4032 | 0.015 | -0.2108 | No |
| 138 | PSME2 |  |  | 4036 | 0.015 | -0.2108 | No |
| 139 | NDRG2 |  |  | 4043 | 0.015 | -0.2112 | No |
| 140 | COG2 |  |  | 4098 | 0.014 | -0.2170 | No |
| 141 | SALL3 |  |  | 4113 | 0.014 | -0.2182 | No |
| 142 | SPRED1 |  |  | 4165 | 0.013 | -0.2237 | No |
| 143 | NBPF14 |  |  | 4258 | 0.011 | -0.2337 | No |
| 144 | HMGCS1 |  |  | 4272 | 0.011 | -0.2350 | No |
| 145 | SNTA1 |  |  | 4285 | 0.011 | -0.2361 | No |
| 146 | IRF2 |  |  | 4326 | 0.010 | -0.2403 | No |
| 147 | PHACTR2 |  |  | 4339 | 0.010 | -0.2415 | No |
| 148 | ENHO |  |  | 4360 | 0.010 | -0.2435 | No |
| 149 | CNDP2 |  |  | 4484 | 0.008 | -0.2571 | No |
| 150 | BDH2 |  |  | 4538 | 0.007 | -0.2629 | No |
| 151 | BEND7 |  |  | 4624 | 0.005 | -0.2723 | No |
| 152 | SPRED2 |  |  | 4630 | 0.005 | -0.2727 | No |
| 153 | VPS36 |  |  | 4649 | 0.005 | -0.2746 | No |
| 154 | VEGFA |  |  | 4721 | 0.004 | -0.2825 | No |
| 155 | SEPT8 |  |  | 4734 | 0.004 | -0.2837 | No |
| 156 | NADK2 |  |  | 4763 | 0.003 | -0.2868 | No |
| 157 | SRPK2 |  |  | 4768 | 0.003 | -0.2872 | No |
| 158 | OLFM2 |  |  | 4808 | 0.003 | -0.2915 | No |
| 159 | HADHB |  |  | 4810 | 0.003 | -0.2915 | No |
| 160 | DGKH |  |  | 4858 | 0.002 | -0.2968 | No |
| 161 | PKM |  |  | 4880 | 0.001 | -0.2991 | No |
| 162 | ROBO3 |  |  | 4933 | 0.001 | -0.3049 | No |
| 163 | KAT2A |  |  | 4974 | 0.000 | -0.3093 | No |
| 164 | SASH1 |  |  | 4986 | 0.000 | -0.3106 | No |
| 165 | SWAP70 |  |  | 5075 | -0.001 | -0.3204 | No |
| 166 | ABCD3 |  |  | 5091 | -0.001 | -0.3220 | No |
| 167 | ATF7 |  |  | 5114 | -0.002 | -0.3244 | No |
| 168 | KCNJ16 |  |  | 5145 | -0.002 | -0.3277 | No |
| 169 | GULP1 |  |  | 5158 | -0.003 | -0.3290 | No |
| 170 | TRAPPC3 |  |  | 5169 | -0.003 | -0.3301 | No |
| 171 | TCF7L2 |  |  | 5199 | -0.003 | -0.3332 | No |
| 172 | PNPT1 |  |  | 5231 | -0.004 | -0.3366 | No |
| 173 | LPIN2 |  |  | 5264 | -0.004 | -0.3401 | No |
| 174 | MTMR10 |  |  | 5281 | -0.005 | -0.3418 | No |
| 175 | NEK1 |  |  | 5385 | -0.007 | -0.3532 | No |
| 176 | SLC38A3 |  |  | 5423 | -0.007 | -0.3572 | No |
| 177 | IQSEC1 |  |  | 5482 | -0.008 | -0.3635 | No |
| 178 | MRPS5 |  |  | 5493 | -0.008 | -0.3644 | No |
| 179 | PDE4B |  |  | 5498 | -0.008 | -0.3647 | No |
| 180 | ZNF678 |  |  | 5552 | -0.009 | -0.3704 | No |
| 181 | TRIM9 |  |  | 5580 | -0.010 | -0.3732 | No |
| 182 | RHOJ |  |  | 5586 | -0.010 | -0.3736 | No |
| 183 | ABLIM1 |  |  | 5605 | -0.010 | -0.3754 | No |
| 184 | AHCYL1 |  |  | 5638 | -0.011 | -0.3788 | No |
| 185 | SIRPA |  |  | 5641 | -0.011 | -0.3788 | No |
| 186 | ALDH7A1 |  |  | 5776 | -0.013 | -0.3935 | No |
| 187 | IFT88 |  |  | 5846 | -0.014 | -0.4009 | No |
| 188 | SCP2 |  |  | 5857 | -0.014 | -0.4017 | No |
| 189 | SSPN |  |  | 5862 | -0.014 | -0.4019 | No |
| 190 | MAGI1 |  |  | 5887 | -0.014 | -0.4043 | No |
| 191 | NACC2 |  |  | 5913 | -0.015 | -0.4068 | No |
| 192 | CHP1 |  |  | 5924 | -0.015 | -0.4076 | No |
| 193 | ARAP2 |  |  | 5936 | -0.015 | -0.4085 | No |
| 194 | PTPN13 |  |  | 5985 | -0.016 | -0.4135 | No |
| 195 | CCDC80 |  |  | 6077 | -0.018 | -0.4233 | No |
| 196 | MCCC2 |  |  | 6082 | -0.018 | -0.4234 | No |
| 197 | FYN |  |  | 6106 | -0.018 | -0.4256 | No |
| 198 | CHKA |  |  | 6122 | -0.019 | -0.4269 | No |
| 199 | HEATR5A |  |  | 6130 | -0.019 | -0.4273 | No |
| 200 | HOPX |  |  | 6156 | -0.019 | -0.4297 | No |
| 201 | ERAP1 |  |  | 6158 | -0.019 | -0.4294 | No |
| 202 | AHCYL2 |  |  | 6171 | -0.019 | -0.4303 | No |
| 203 | HES1 |  |  | 6191 | -0.020 | -0.4321 | No |
| 204 | IDH1 |  |  | 6208 | -0.020 | -0.4334 | No |
| 205 | CAMK2G |  |  | 6251 | -0.021 | -0.4377 | No |
| 206 | BMPR1A |  |  | 6281 | -0.021 | -0.4405 | No |
| 207 | MED13 |  |  | 6295 | -0.022 | -0.4415 | No |
| 208 | SLC35A5 |  |  | 6314 | -0.022 | -0.4431 | No |
| 209 | SNAP23 |  |  | 6326 | -0.022 | -0.4439 | No |
| 210 | TSPAN7 |  |  | 6363 | -0.023 | -0.4474 | No |
| 211 | DOCK1 |  |  | 6381 | -0.023 | -0.4488 | No |
| 212 | TEAD1 |  |  | 6417 | -0.024 | -0.4523 | No |
| 213 | ARHGAP31 |  |  | 6431 | -0.024 | -0.4532 | No |
| 214 | FUT11 |  |  | 6436 | -0.024 | -0.4532 | No |
| 215 | PTPRD |  |  | 6440 | -0.024 | -0.4530 | No |
| 216 | ILDR2 |  |  | 6458 | -0.025 | -0.4544 | No |
| 217 | NLGN3 |  |  | 6477 | -0.025 | -0.4559 | No |
| 218 | TMEM170A |  |  | 6509 | -0.025 | -0.4589 | No |
| 219 | MLLT6 |  |  | 6557 | -0.026 | -0.4636 | No |
| 220 | EEPD1 |  |  | 6558 | -0.026 | -0.4630 | No |
| 221 | ABAT |  |  | 6574 | -0.027 | -0.4642 | No |
| 222 | PAQR8 |  |  | 6579 | -0.027 | -0.4641 | No |
| 223 | SAMD8 |  |  | 6620 | -0.028 | -0.4680 | No |
| 224 | DPM3 |  |  | 6664 | -0.029 | -0.4722 | No |
| 225 | ZFYVE16 |  |  | 6723 | -0.030 | -0.4780 | No |
| 226 | BBS2 |  |  | 6732 | -0.030 | -0.4783 | No |
| 227 | MYO10 |  |  | 6758 | -0.031 | -0.4805 | No |
| 228 | APOL2 |  |  | 6764 | -0.031 | -0.4804 | No |
| 229 | ABI1 |  |  | 6766 | -0.031 | -0.4799 | No |
| 230 | AGPAT3 |  |  | 6767 | -0.031 | -0.4793 | No |
| 231 | STK3 |  |  | 6785 | -0.031 | -0.4805 | No |
| 232 | METTL8 |  |  | 6792 | -0.031 | -0.4806 | No |
| 233 | SLC18B1 |  |  | 6813 | -0.032 | -0.4822 | No |
| 234 | PARP9 |  |  | 6819 | -0.032 | -0.4821 | No |
| 235 | LYPD1 |  |  | 6876 | -0.033 | -0.4876 | No |
| 236 | SLC35F1 |  |  | 6914 | -0.035 | -0.4911 | No |
| 237 | JAM2 |  |  | 6939 | -0.035 | -0.4930 | No |
| 238 | LRRN1 |  |  | 6981 | -0.036 | -0.4969 | No |
| 239 | PIK3R1 |  |  | 6997 | -0.036 | -0.4978 | No |
| 240 | DPP8 |  |  | 7023 | -0.037 | -0.4998 | No |
| 241 | ZNF521 |  |  | 7041 | -0.038 | -0.5010 | No |
| 242 | ZDHHC21 |  |  | 7047 | -0.038 | -0.5008 | No |
| 243 | ARHGAP12 |  |  | 7074 | -0.038 | -0.5029 | No |
| 244 | LRP4 |  |  | 7084 | -0.039 | -0.5031 | No |
| 245 | PSENEN |  |  | 7090 | -0.039 | -0.5029 | No |
| 246 | TOB2 |  |  | 7133 | -0.040 | -0.5067 | No |
| 247 | NOTCH3 |  |  | 7196 | -0.042 | -0.5128 | No |
| 248 | BBX |  |  | 7215 | -0.042 | -0.5140 | No |
| 249 | FOS |  |  | 7222 | -0.043 | -0.5138 | No |
| 250 | TNFRSF19 |  |  | 7226 | -0.043 | -0.5132 | No |
| 251 | ZC3HAV1 |  |  | 7233 | -0.043 | -0.5130 | No |
| 252 | PTCH1 |  |  | 7258 | -0.044 | -0.5148 | No |
| 253 | ABCA3 |  |  | 7275 | -0.044 | -0.5157 | No |
| 254 | PCDH17 |  |  | 7304 | -0.045 | -0.5179 | No |
| 255 | KANK1 |  |  | 7308 | -0.045 | -0.5173 | No |
| 256 | DPF3 |  |  | 7344 | -0.046 | -0.5203 | No |
| 257 | SPATS2L |  |  | 7352 | -0.046 | -0.5201 | No |
| 258 | CSPG5 |  |  | 7377 | -0.047 | -0.5219 | No |
| 259 | FMNL2 |  |  | 7409 | -0.047 | -0.5244 | No |
| 260 | LAPTM4B |  |  | 7427 | -0.048 | -0.5253 | No |
| 261 | GABBR1 |  |  | 7437 | -0.048 | -0.5253 | No |
| 262 | IVD |  |  | 7438 | -0.048 | -0.5243 | No |
| 263 | QKI |  |  | 7478 | -0.050 | -0.5277 | No |
| 264 | ZNF236 |  |  | 7498 | -0.050 | -0.5288 | No |
| 265 | ID1 |  |  | 7519 | -0.051 | -0.5300 | No |
| 266 | RNPC3 |  |  | 7532 | -0.051 | -0.5303 | No |
| 267 | CTNNB1 |  |  | 7580 | -0.053 | -0.5344 | No |
| 268 | CERS6 |  |  | 7583 | -0.053 | -0.5336 | No |
| 269 | NCKAP5 |  |  | 7602 | -0.053 | -0.5345 | No |
| 270 | ATP9B |  |  | 7608 | -0.054 | -0.5340 | No |
| 271 | PARP14 |  |  | 7641 | -0.054 | -0.5364 | No |
| 272 | F3 |  |  | 7662 | -0.055 | -0.5375 | No |
| 273 | PDLIM5 |  |  | 7663 | -0.055 | -0.5364 | No |
| 274 | TAOK3 |  |  | 7664 | -0.055 | -0.5353 | No |
| 275 | MCC |  |  | 7673 | -0.055 | -0.5351 | No |
| 276 | NDRG4 |  |  | 7675 | -0.055 | -0.5341 | No |
| 277 | IFNAR1 |  |  | 7683 | -0.056 | -0.5337 | No |
| 278 | CASC4 |  |  | 7687 | -0.056 | -0.5329 | No |
| 279 | HS2ST1 |  |  | 7729 | -0.057 | -0.5363 | No |
| 280 | SLC25A18 |  |  | 7731 | -0.057 | -0.5353 | No |
| 281 | KAT2B |  |  | 7738 | -0.058 | -0.5348 | No |
| 282 | CLDND1 |  |  | 7767 | -0.059 | -0.5367 | No |
| 283 | DNER |  |  | 7777 | -0.059 | -0.5365 | No |
| 284 | MEIS1 |  |  | 7785 | -0.059 | -0.5361 | No |
| 285 | NPAS3 |  |  | 7814 | -0.060 | -0.5380 | No |
| 286 | RASSF4 |  |  | 7851 | -0.061 | -0.5407 | No |
| 287 | LDLRAD3 |  |  | 7860 | -0.062 | -0.5404 | No |
| 288 | GLDC |  |  | 7897 | -0.063 | -0.5431 | No |
| 289 | SC5D |  |  | 7900 | -0.063 | -0.5421 | No |
| 290 | PTAR1 |  |  | 7908 | -0.063 | -0.5416 | No |
| 291 | NLGN4X |  |  | 7947 | -0.065 | -0.5445 | No |
| 292 | LPP |  |  | 7981 | -0.066 | -0.5468 | No |
| 293 | ST3GAL5 |  |  | 7982 | -0.066 | -0.5455 | No |
| 294 | ARHGEF6 |  |  | 7988 | -0.067 | -0.5447 | No |
| 295 | GATM |  |  | 7990 | -0.067 | -0.5434 | No |
| 296 | ZNF621 |  |  | 8017 | -0.068 | -0.5449 | No |
| 297 | HIBCH |  |  | 8030 | -0.068 | -0.5449 | No |
| 298 | PCDH9 |  |  | 8035 | -0.068 | -0.5439 | No |
| 299 | SDC3 |  |  | 8064 | -0.070 | -0.5456 | No |
| 300 | PCDHGC3 |  |  | 8091 | -0.071 | -0.5471 | No |
| 301 | EGFR |  |  | 8094 | -0.071 | -0.5459 | No |
| 302 | S100A16 |  |  | 8127 | -0.073 | -0.5480 | No |
| 303 | LIFR |  |  | 8134 | -0.073 | -0.5471 | No |
| 304 | PMP2 |  |  | 8160 | -0.075 | -0.5484 | No |
| 305 | PLEKHG1 |  |  | 8179 | -0.076 | -0.5488 | Yes |
| 306 | STK17A |  |  | 8190 | -0.077 | -0.5484 | Yes |
| 307 | SSR3 |  |  | 8191 | -0.077 | -0.5468 | Yes |
| 308 | AASS |  |  | 8209 | -0.078 | -0.5472 | Yes |
| 309 | RFX4 |  |  | 8211 | -0.078 | -0.5457 | Yes |
| 310 | PTPRA |  |  | 8219 | -0.078 | -0.5449 | Yes |
| 311 | DCLK2 |  |  | 8232 | -0.079 | -0.5446 | Yes |
| 312 | SPPL2A |  |  | 8249 | -0.080 | -0.5448 | Yes |
| 313 | IFI6 |  |  | 8257 | -0.080 | -0.5439 | Yes |
| 314 | ZMAT1 |  |  | 8261 | -0.081 | -0.5426 | Yes |
| 315 | UFL1 |  |  | 8281 | -0.082 | -0.5431 | Yes |
| 316 | NLGN1 |  |  | 8288 | -0.082 | -0.5421 | Yes |
| 317 | GLUL |  |  | 8307 | -0.083 | -0.5424 | Yes |
| 318 | SFXN5 |  |  | 8309 | -0.083 | -0.5408 | Yes |
| 319 | PBXIP1 |  |  | 8337 | -0.085 | -0.5421 | Yes |
| 320 | SPECC1 |  |  | 8346 | -0.085 | -0.5412 | Yes |
| 321 | NOTCH2 |  |  | 8358 | -0.086 | -0.5407 | Yes |
| 322 | SLC4A4 |  |  | 8359 | -0.086 | -0.5390 | Yes |
| 323 | HEPACAM |  |  | 8380 | -0.088 | -0.5394 | Yes |
| 324 | PLOD2 |  |  | 8403 | -0.089 | -0.5400 | Yes |
| 325 | RFTN2 |  |  | 8418 | -0.090 | -0.5398 | Yes |
| 326 | SEL1L3 |  |  | 8436 | -0.091 | -0.5398 | Yes |
| 327 | FADS2 |  |  | 8492 | -0.095 | -0.5440 | Yes |
| 328 | ATP1B2 |  |  | 8494 | -0.095 | -0.5422 | Yes |
| 329 | PDGFRA |  |  | 8503 | -0.096 | -0.5411 | Yes |
| 330 | CTNND2 |  |  | 8509 | -0.097 | -0.5397 | Yes |
| 331 | MCFD2 |  |  | 8523 | -0.098 | -0.5392 | Yes |
| 332 | ACOX1 |  |  | 8525 | -0.098 | -0.5373 | Yes |
| 333 | ADAMTS6 |  |  | 8530 | -0.099 | -0.5357 | Yes |
| 334 | MEST |  |  | 8534 | -0.099 | -0.5340 | Yes |
| 335 | APC |  |  | 8542 | -0.100 | -0.5328 | Yes |
| 336 | BMP7 |  |  | 8549 | -0.100 | -0.5314 | Yes |
| 337 | MYO6 |  |  | 8550 | -0.100 | -0.5294 | Yes |
| 338 | HEY1 |  |  | 8555 | -0.100 | -0.5278 | Yes |
| 339 | GPC6 |  |  | 8558 | -0.100 | -0.5260 | Yes |
| 340 | LRP1B |  |  | 8564 | -0.101 | -0.5245 | Yes |
| 341 | MFGE8 |  |  | 8573 | -0.101 | -0.5233 | Yes |
| 342 | SEMA6A |  |  | 8574 | -0.101 | -0.5212 | Yes |
| 343 | COL11A1 |  |  | 8598 | -0.103 | -0.5217 | Yes |
| 344 | ITM2C |  |  | 8600 | -0.103 | -0.5197 | Yes |
| 345 | CAMK2D |  |  | 8602 | -0.103 | -0.5177 | Yes |
| 346 | CSGALNACT1 |  |  | 8615 | -0.104 | -0.5169 | Yes |
| 347 | CADM2 |  |  | 8653 | -0.107 | -0.5189 | Yes |
| 348 | TMEM9B |  |  | 8658 | -0.108 | -0.5171 | Yes |
| 349 | LGALS1 |  |  | 8680 | -0.110 | -0.5172 | Yes |
| 350 | VIM |  |  | 8691 | -0.110 | -0.5161 | Yes |
| 351 | CST3 |  |  | 8712 | -0.112 | -0.5160 | Yes |
| 352 | RGS20 |  |  | 8714 | -0.112 | -0.5139 | Yes |
| 353 | FGFR3 |  |  | 8715 | -0.112 | -0.5116 | Yes |
| 354 | GJA1 |  |  | 8728 | -0.114 | -0.5106 | Yes |
| 355 | ZMYM2 |  |  | 8733 | -0.115 | -0.5087 | Yes |
| 356 | RNF180 |  |  | 8736 | -0.115 | -0.5066 | Yes |
| 357 | TRIM47 |  |  | 8738 | -0.115 | -0.5044 | Yes |
| 358 | MEGF10 |  |  | 8741 | -0.115 | -0.5022 | Yes |
| 359 | MLEC |  |  | 8744 | -0.116 | -0.5001 | Yes |
| 360 | LRRN3 |  |  | 8753 | -0.117 | -0.4986 | Yes |
| 361 | NOTCH1 |  |  | 8763 | -0.118 | -0.4972 | Yes |
| 362 | BCHE |  |  | 8775 | -0.119 | -0.4960 | Yes |
| 363 | ID2 |  |  | 8781 | -0.120 | -0.4942 | Yes |
| 364 | NTM |  |  | 8787 | -0.120 | -0.4923 | Yes |
| 365 | RGMA |  |  | 8807 | -0.123 | -0.4919 | Yes |
| 366 | CPE |  |  | 8816 | -0.125 | -0.4902 | Yes |
| 367 | ITGA6 |  |  | 8820 | -0.125 | -0.4880 | Yes |
| 368 | DTNA |  |  | 8828 | -0.126 | -0.4862 | Yes |
| 369 | DAD1 |  |  | 8847 | -0.128 | -0.4856 | Yes |
| 370 | PLEKHH2 |  |  | 8850 | -0.129 | -0.4832 | Yes |
| 371 | SEZ6L |  |  | 8858 | -0.130 | -0.4813 | Yes |
| 372 | OPHN1 |  |  | 8872 | -0.133 | -0.4801 | Yes |
| 373 | HSD17B12 |  |  | 8878 | -0.133 | -0.4779 | Yes |
| 374 | S100A13 |  |  | 8880 | -0.133 | -0.4753 | Yes |
| 375 | DNASE2 |  |  | 8885 | -0.133 | -0.4731 | Yes |
| 376 | MMP16 |  |  | 8897 | -0.135 | -0.4715 | Yes |
| 377 | CYR61 |  |  | 8901 | -0.136 | -0.4691 | Yes |
| 378 | TGFB2 |  |  | 8921 | -0.137 | -0.4684 | Yes |
| 379 | RNF13 |  |  | 8922 | -0.137 | -0.4656 | Yes |
| 380 | ITPR2 |  |  | 8925 | -0.138 | -0.4630 | Yes |
| 381 | RCN1 |  |  | 8929 | -0.138 | -0.4606 | Yes |
| 382 | DSEL |  |  | 8943 | -0.140 | -0.4592 | Yes |
| 383 | GPRC5B |  |  | 8956 | -0.142 | -0.4576 | Yes |
| 384 | FGFBP3 |  |  | 8960 | -0.143 | -0.4550 | Yes |
| 385 | TAPBP |  |  | 8966 | -0.144 | -0.4526 | Yes |
| 386 | TRPS1 |  |  | 8967 | -0.144 | -0.4497 | Yes |
| 387 | SLC16A1 |  |  | 8970 | -0.145 | -0.4470 | Yes |
| 388 | SCD |  |  | 8982 | -0.146 | -0.4452 | Yes |
| 389 | NTRK3 |  |  | 8986 | -0.146 | -0.4426 | Yes |
| 390 | TXNIP |  |  | 8987 | -0.147 | -0.4396 | Yes |
| 391 | PTN |  |  | 8989 | -0.147 | -0.4367 | Yes |
| 392 | PPIB |  |  | 8991 | -0.147 | -0.4338 | Yes |
| 393 | JAG1 |  |  | 8993 | -0.148 | -0.4309 | Yes |
| 394 | SEMA6D |  |  | 8994 | -0.148 | -0.4279 | Yes |
| 395 | TNK2 |  |  | 8997 | -0.149 | -0.4251 | Yes |
| 396 | ADCY2 |  |  | 9004 | -0.149 | -0.4227 | Yes |
| 397 | MTRNR2L8 |  |  | 9018 | -0.152 | -0.4211 | Yes |
| 398 | AXL |  |  | 9023 | -0.152 | -0.4184 | Yes |
| 399 | MAML2 |  |  | 9028 | -0.154 | -0.4157 | Yes |
| 400 | TTYH1 |  |  | 9029 | -0.154 | -0.4126 | Yes |
| 401 | PON2 |  |  | 9030 | -0.154 | -0.4094 | Yes |
| 402 | TMEM106B |  |  | 9036 | -0.155 | -0.4068 | Yes |
| 403 | COMT |  |  | 9039 | -0.156 | -0.4039 | Yes |
| 404 | SLC7A11 |  |  | 9040 | -0.156 | -0.4007 | Yes |
| 405 | NRBP2 |  |  | 9048 | -0.157 | -0.3983 | Yes |
| 406 | SLC1A3 |  |  | 9052 | -0.158 | -0.3954 | Yes |
| 407 | SERPINH1 |  |  | 9054 | -0.159 | -0.3923 | Yes |
| 408 | CHPT1 |  |  | 9057 | -0.159 | -0.3892 | Yes |
| 409 | LRRC17 |  |  | 9058 | -0.159 | -0.3860 | Yes |
| 410 | TFPI |  |  | 9060 | -0.159 | -0.3829 | Yes |
| 411 | PRNP |  |  | 9068 | -0.161 | -0.3804 | Yes |
| 412 | IL6ST |  |  | 9090 | -0.164 | -0.3794 | Yes |
| 413 | LGALS3BP |  |  | 9114 | -0.169 | -0.3785 | Yes |
| 414 | PNISR |  |  | 9119 | -0.170 | -0.3755 | Yes |
| 415 | PTPRG |  |  | 9127 | -0.172 | -0.3727 | Yes |
| 416 | CADM4 |  |  | 9128 | -0.172 | -0.3692 | Yes |
| 417 | ALCAM |  |  | 9132 | -0.173 | -0.3661 | Yes |
| 418 | SCD5 |  |  | 9133 | -0.174 | -0.3625 | Yes |
| 419 | PRCP |  |  | 9153 | -0.178 | -0.3610 | Yes |
| 420 | MSMO1 |  |  | 9162 | -0.179 | -0.3582 | Yes |
| 421 | HTRA1 |  |  | 9178 | -0.184 | -0.3562 | Yes |
| 422 | APCDD1 |  |  | 9180 | -0.185 | -0.3525 | Yes |
| 423 | DPP6 |  |  | 9181 | -0.185 | -0.3487 | Yes |
| 424 | SYPL1 |  |  | 9182 | -0.185 | -0.3450 | Yes |
| 425 | SERPINE2 |  |  | 9186 | -0.186 | -0.3415 | Yes |
| 426 | GOLIM4 |  |  | 9189 | -0.187 | -0.3379 | Yes |
| 427 | FAM3C |  |  | 9190 | -0.187 | -0.3341 | Yes |
| 428 | DAG1 |  |  | 9192 | -0.187 | -0.3304 | Yes |
| 429 | TMED10 |  |  | 9196 | -0.189 | -0.3269 | Yes |
| 430 | SYT11 |  |  | 9197 | -0.189 | -0.3231 | Yes |
| 431 | TTC14 |  |  | 9198 | -0.190 | -0.3192 | Yes |
| 432 | SCRG1 |  |  | 9200 | -0.191 | -0.3154 | Yes |
| 433 | TMEM132B |  |  | 9205 | -0.192 | -0.3119 | Yes |
| 434 | FAT1 |  |  | 9207 | -0.193 | -0.3081 | Yes |
| 435 | FKBP10 |  |  | 9215 | -0.199 | -0.3048 | Yes |
| 436 | CD99 |  |  | 9224 | -0.203 | -0.3016 | Yes |
| 437 | LRIG1 |  |  | 9229 | -0.204 | -0.2979 | Yes |
| 438 | IGFBP2 |  |  | 9240 | -0.208 | -0.2948 | Yes |
| 439 | CLSTN1 |  |  | 9246 | -0.210 | -0.2911 | Yes |
| 440 | ABHD2 |  |  | 9250 | -0.213 | -0.2870 | Yes |
| 441 | TMBIM6 |  |  | 9264 | -0.219 | -0.2840 | Yes |
| 442 | SESN3 |  |  | 9266 | -0.219 | -0.2797 | Yes |
| 443 | LRRC4C |  |  | 9267 | -0.220 | -0.2752 | Yes |
| 444 | MLC1 |  |  | 9269 | -0.220 | -0.2708 | Yes |
| 445 | LSAMP |  |  | 9273 | -0.221 | -0.2667 | Yes |
| 446 | ZMAT3 |  |  | 9276 | -0.223 | -0.2623 | Yes |
| 447 | SAT1 |  |  | 9279 | -0.224 | -0.2580 | Yes |
| 448 | FJX1 |  |  | 9282 | -0.225 | -0.2537 | Yes |
| 449 | NTRK2 |  |  | 9299 | -0.230 | -0.2508 | Yes |
| 450 | SEMA5A |  |  | 9315 | -0.243 | -0.2475 | Yes |
| 451 | LAMB2 |  |  | 9319 | -0.246 | -0.2428 | Yes |
| 452 | CD9 |  |  | 9324 | -0.248 | -0.2382 | Yes |
| 453 | STAT3 |  |  | 9325 | -0.249 | -0.2331 | Yes |
| 454 | HSP90B1 |  |  | 9334 | -0.257 | -0.2288 | Yes |
| 455 | LRP1 |  |  | 9338 | -0.259 | -0.2238 | Yes |
| 456 | ZBTB20 |  |  | 9341 | -0.261 | -0.2187 | Yes |
| 457 | DDR1 |  |  | 9345 | -0.266 | -0.2136 | Yes |
| 458 | ITGAV |  |  | 9346 | -0.269 | -0.2082 | Yes |
| 459 | PMP22 |  |  | 9347 | -0.271 | -0.2026 | Yes |
| 460 | NOVA1 |  |  | 9348 | -0.272 | -0.1971 | Yes |
| 461 | SLC2A1 |  |  | 9349 | -0.272 | -0.1916 | Yes |
| 462 | PTPRZ1 |  |  | 9351 | -0.272 | -0.1861 | Yes |
| 463 | BCAN |  |  | 9353 | -0.276 | -0.1806 | Yes |
| 464 | CD81 |  |  | 9356 | -0.281 | -0.1751 | Yes |
| 465 | SPARCL1 |  |  | 9360 | -0.288 | -0.1696 | Yes |
| 466 | ITGB8 |  |  | 9362 | -0.294 | -0.1637 | Yes |
| 467 | PSAP |  |  | 9363 | -0.294 | -0.1577 | Yes |
| 468 | EDNRB |  |  | 9369 | -0.301 | -0.1522 | Yes |
| 469 | CANX |  |  | 9374 | -0.310 | -0.1463 | Yes |
| 470 | TIMP2 |  |  | 9379 | -0.316 | -0.1403 | Yes |
| 471 | GPM6A |  |  | 9381 | -0.324 | -0.1338 | Yes |
| 472 | B2M |  |  | 9383 | -0.331 | -0.1272 | Yes |
| 473 | TIMP1 |  |  | 9387 | -0.334 | -0.1207 | Yes |
| 474 | GPM6B |  |  | 9389 | -0.344 | -0.1138 | Yes |
| 475 | WSB1 |  |  | 9393 | -0.350 | -0.1070 | Yes |
| 476 | CD63 |  |  | 9394 | -0.353 | -0.0998 | Yes |
| 477 | ITM2B |  |  | 9399 | -0.371 | -0.0927 | Yes |
| 478 | TNC |  |  | 9403 | -0.383 | -0.0852 | Yes |
| 479 | COL6A1 |  |  | 9410 | -0.410 | -0.0776 | Yes |
| 480 | DST |  |  | 9422 | -0.485 | -0.0689 | Yes |
| 481 | GFAP |  |  | 9425 | -0.504 | -0.0589 | Yes |
| 482 | CLU |  |  | 9430 | -0.547 | -0.0482 | Yes |
| 483 | MALAT1 |  |  | 9436 | -0.735 | -0.0338 | Yes |
| 484 | IGFBP5 |  |  | 9438 | -0.797 | -0.0176 | Yes |
| 485 | NEAT1 |  |  | 9439 | -0.871 | 0.0001 | Yes |
Table: GSEA details [plain text format]

  

Fig 2: RICHARDS\_NOWAKOWSKI\_SCIENCE\_2017\_ASTROCYTE\_UPREG: Random ES distribution      
 Gene set null distribution of ES for **RICHARDS\_NOWAKOWSKI\_SCIENCE\_2017\_ASTROCYTE\_UPREG**
